# A novel fungal metabolite inhibits *Plasmodium falciparum* transmission and infection

**DOI:** 10.1186/s13071-021-04677-7

**Published:** 2021-03-24

**Authors:** Guodong Niu, Xiaohong Wang, Yue Hao, Shambhu Kandel, Guomin Niu, Raphael G. Raptis, Jun Li

**Affiliations:** 1 Department of Biological Sciences, Florida International University, 11200 SW 8th St, Miami, FL 33199 USA; 2 Department of Chemistry and Biochemistry, Florida International University, 11200 SW 8th St, Miami, FL 33199 USA; 3 Biomolecular Sciences Institute, Florida International University, 11200 SW 8th St, Miami, FL 33199 USA; 4 Department of Hematology, Southern Medical University Affiliated Nanhai Hospital, Guangzhou, Guangdong China; 5 College of Public Health, University of South China, Hengyang, Hunan China

**Keywords:** Fungal metabolites, Antimalarial agent, Malaria, Mosquito, FREP1-mediated *Plasmodium* transmission, *Purpureocillium lilacinum*

## Abstract

**Background:**

Malaria transmission depends on infected mosquitoes and can be controlled by transmission-blocking drugs. The recently discovered FREP1-mediated malaria transmission pathway is an excellent target to screen drugs for limiting transmission.

**Methods:**

To identify candidate small molecules, we used an ELISA-based approach to analyze extracts from a fungal library for inhibition of the FREP1–parasite interaction. We isolated and determined one active compound by chromatography and crystallography, respectively. We measured the effects of the bioactive compound on malaria transmission to mosquitoes through standard membrane-feeding assays (SMFA) and on parasite proliferation in blood by culturing.

**Results:**

We discovered the ethyl acetate extract of the fungus *Purpureocillium lilacinum* that inhibited *Plasmodium falciparum* transmission to mosquitoes. Pre-exposure to the extract rendered *Anopheles gambiae* resistant to *Plasmodium* infection. Furthermore, we isolated one novel active compound from the extract and identified it as 3-amino-7,9-dihydroxy-1-methyl-6H-benzo[c]chromen-6-one, or “pulixin.” Pulixin prevented FREP1 from binding to *P. falciparum*-infected cell lysate. Pulixin blocked the transmission of the parasite to mosquitoes with an EC_50_ (the concentration that gave half-maximal response) of 11 µM based on SMFA. Notably, pulixin also inhibited the proliferation of asexual-stage *P. falciparum* with an EC_50_ of 47 nM. The compound did not show cytotoxic effects at a concentration of 116 µM or lower.

**Conclusion:**

By targeting the FREP1–*Plasmodium* interaction, we discovered that *Purpureocillium lilacinum* extract blocked malaria transmission. We isolated and identified the bioactive agent pulixin as a new compound capable of stopping malaria transmission to mosquitoes and inhibiting parasite proliferation in blood culture.
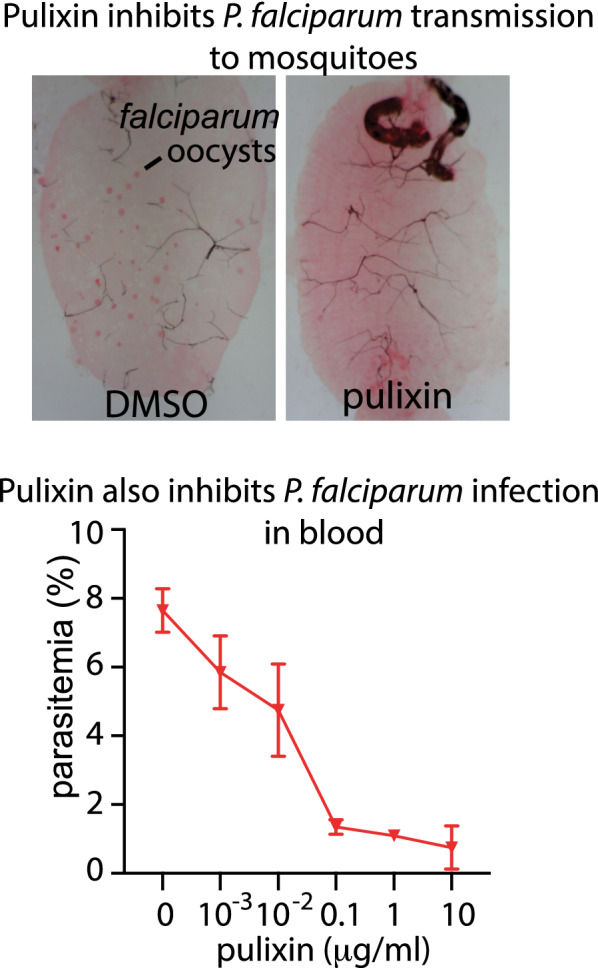

**Supplementary Information:**

The online version contains supplementary material available at 10.1186/s13071-021-04677-7.

## Background

*Plasmodium* parasites transmitted by anopheline mosquitoes caused approximately 200 million clinical malaria cases and half a million deaths in 2019, according to a recent report by the World Health Organization (WHO) [1]. Most antimalarial drugs kill the parasites at the blood stage [2–4]. Since the passage of *Plasmodium* through vector mosquitoes is a necessary step for malaria transmission, using insecticides to control the mosquito population has traditionally been an effective method for preventing the disease. However, the spread of insecticide resistance [5] in mosquito populations [6, 7] and the lack of vaccines against the disease have prompted the public health community to advocate new strategies for malaria control.

This study focuses on the discovery of small molecules that interrupt malaria transmission. During malaria transmission from a host to mosquitoes, mosquito proteins such as Tep1, APL1C, and LRIM1 inhibit *Plasmodium* infection of mosquitoes [8–10], while other mosquito proteins such as the fibrinogen-related protein 1 (FREP1) facilitate *Plasmodium* invasion. FREP1 binds to parasites in the mosquito midgut and mediates *Plasmodium* invasion [11, 12]. Antibodies against FREP1 inhibit infection by *P. vivax*, *P. falciparum*, and *P. berghei* of *Anopheles dirus* and *An. gambiae* mosquitoes [12, 13], supporting the hypothesis that this pathway is conserved across multiple *Plasmodium* and *Anopheles species*. FREP1 belongs to the fibrinogen-related protein family, whose members contain a conserved fibrinogen-like (FBG) domain with approximately 200 amino acids [14, 15]. In mammals, fibrinogens are involved in blood coagulation, whereas in invertebrates, they function as pattern recognition receptors capable of binding to bacteria, fungi, or parasites [16]. Since mosquito FREP1 facilitates *Plasmodium* infection through direct binding to gametocytes and ookinetes [12], small molecules that interrupt this interaction should be ideal candidates to block malaria transmission [17, 18]. Such compounds can be administered to malaria patients or be sprayed outdoors, indoors, or on bed nets. At present, very few preparations are available in the market for this purpose [19].

Here we screened our global fungal library with more than 10,000 different fungal strains [20] and identified one novel compound that inhibited *P. falciparum* proliferation and transmission.

## Methods

### Screening the fungal extract library to discover malaria transmission-blocking candidates

As described previously [18], we screened small molecules that inhibited the FREP1–*Plasmodium* interaction to block malaria transmission. In brief, *P. falciparum*-infected (NF54 obtained from the BEI Resources Repository, Manassas, VA, USA) red blood cells (iRBCs) were cultured for 15–17 days. The iRBCs suspended in PBST (PBS containing 0.2% Tween-20) were homogenized by ultrasonication with six cycles of 10 s of pulse and 50 s of resting on ice for each period. The lysates were centrifuged at 8000×*g* for 2 min to remove insoluble materials and cellular debris. Then, 96-well enzyme-linked immunosorbent assay (ELISA) plates were coated with 50 μL of the iRBC lysate (2 mg/mL protein) overnight at 4 °C. After coating, the wells were blocked with 100 μL of 2% bovine serum albumin (BSA) in PBS per well for 1.5 h at room temperature (RT). After removal of the blocking solution, 49 μL of FREP1 (10 μg/mL) [12] in blocking buffer (PBS containing 2% BSA) and 1 μL of fungal extract (2 mg/mL in DMSO) were added to each well, followed by incubation for 1 h at RT. The wells were washed three times with 100 μL PBST, and 50 μL of rabbit anti-FREP1 polyclonal antibody [12] (1:5,000 dilution in blocking buffer, ~ 1 µg/mL as the final concentration) was added to each well and incubated for 1 h at RT. About 50 μL of alkaline phosphatase-conjugated anti-rabbit IgG (Sigma-Aldrich, St. Louis, MO, USA; diluted 1:20,000 in blocking buffer) was added to each well and incubated for 45 min at RT. The wells were washed three times with 100 μL PBST between incubations. Finally, each well was developed with 50 μL of pNPP substrate (Sigma-Aldrich) until the colors were visible, and absorbance at 405 nm was measured. The active recombinant FREP1 supplemented with 1 μL of DMSO was the non-inhibition control, and the heat-inactivated recombinant FREP1 (65 °C for 15 min) was the negative control. The following equation was used to calculate the inhibition rate of FREP1–parasite interaction: (A_405_ of DMSO − A_405_ of experimental treatment)/(A_405_ of DMSO − A_405_ of inactivated FREP1).

### Determination of the activity of pulixin in limiting FREP1–parasite interaction

The 15- to 17-day cultured *P. falciparum* infected cell lysate was prepared as described above. The 96-well ELISA plates were coated with 50 μL of the iRBC lysate (2 mg/mL protein) overnight at 4 °C, and the FREP1 protein in PBS (10 μg/mL), together with 0, 2.5, 5, or 10 µg/mL of pulixin, was added to wells and incubated. Rabbit anti-FREP1 polyclonal antibodies quantified the retained FREP1 as described above. After reaction with the pNPP, A_405_ was measured. The assays were conducted in triplicate at each concentration, and the experiments were conducted twice independently.

### Determination of the transmission-blocking activity of the fungal extracts and pure pulixin

The 15- to 17-day-old cultured *P. falciparum* iRBCs containing 2–3% stage V gametocytes were collected and diluted with new O+ type human blood, with the same volume of heat-inactivated AB+ human serum added. The final concentration of stage V gametocytes in the blood was around 0.2%. Then, 3 μL of the candidate fungal extract or pulixin with different concentrations in DMSO was mixed with 297 μL of infected blood and was used to feed about 100 3- to 5-day-old *An. gambiae* G3 female mosquitoes for 30 min, and the engorged mosquitoes were maintained with 8% sugar in a BSL-2 insectary (28 °C, 12 h light/dark cycle, 80% humidity). The midguts were dissected 7 days after infection and stained with 0.1% mercury dibromofluorescein disodium salt in PBS. The oocysts were counted under a light microscope.

### Determination of fungal species

The nuclear ribosomal internal transcribed spacer (ITS) region was amplified with ITS1F (5′-CTTGGTCATTTAGAGGAAGTAA-3′) and ITS4 (5′-TCCTCCGCTTATTGATATGC-3′) primers using the following approach: initial denaturation at 94 °C for 2 min, 35 cycles of denaturation at 94 °C for 30 s, annealing at 55 °C for 30 s, and extension at 72 °C for 1 min, followed by final extension at 72 °C for 5 min. The amplified product was sequenced using the Sanger approach. Original sequences were searched against GenBank using BLAST to determine the fungal species. Alignments for the ITS locus were carried out in MAFFT v7.307 online version [21] and checked visually and modified manually. A maximum parsimony analysis was performed in PAUP* version 4.0b10 [22]. The morphology of the fungi was examined under a microscope (Nikon, Tokyo, Japan). Colonies for observation were grown on potato dextrose agar (PDA) medium plates for 7–15 days at 25 °C.

### Extraction, isolation, and purification of active antimalarial drug candidates

About 500 g Cheerios breakfast cereal (General Mills, Minneapolis, MN, USA) on an open tray was autoclaved with a cycle of 20 min sterilization and 30 min dry time. The sterile cereal was put into a mushroom bag. Two liters of sterile 0.3% sucrose solution containing 0.005% chloramphenicol was added, followed by the inoculation of the candidate fungus. The fungus was cultured at RT for 18 days, and then soaked in the same volume of ethyl acetate overnight. The supernatant was filtered using a Büchner funnel and dried using a rotary evaporator (Heidolph, Elk Grove Village, IL, USA).

The crude extract in methanol was applied onto preparative 60 * 100 mm-GF_254_ silica gel thin-layer chromatography (TLC) plates (Kaibang Separation Materials LLC, Qingdao, China) and separated with a methanol/dichloromethane mixture (1:9 by v/v), and the fluorescence bands were detected at 365 nm and the absorbance bands at 254 nm using a UV-Vis chromatogram analyzer (YUSHEN Instrument Co., Ltd, Shanghai, China). Each band was cut and extracted using 100% methanol. The fractions were dried completely using a rotary evaporator followed by a vacuum oven. The fractions dissolved in DMSO were analyzed by standard membrane-feeding assay (SMFA) for their transmission-blocking activity. The active fraction was subjected to a Shimadzu high-performance liquid chromatography (HPLC) system that included an LC-20AD pump, an SPD-20A UV–Vis detector, and an FRC-10A fraction collector (Columbia, MD, USA) with a Gemini column (5 µm C18 110 Å, 250 mm × 10 mm, Phenomenex, Torrance, CA, USA) to purify and evaluate the purity and characterization of the compound using a gradient solvent of methanol–H_2_O (50:50–100:0).

### Characterization of chemical constituents and structure

The structure of pulixin was determined by X-ray crystallography. Colorless crystals were obtained by slow evaporation of pulixin in the DMSO solution. Single-crystal X-ray data were collected at 295 K using Mo-Κα radiation on a Bruker D8 Quest diffractometer equipped with a CMOS detector. The structure was confirmed by spectroscopic methods, including ^1^H nuclear magnetic resonance (NMR), ^13^C NMR, and electrospray ionization mass spectrometry (ESI–MS). ^1^H NMR spectra were recorded on a Bruker NMR (400 MHz) spectrometer (Bruker Scientific LLC, Billerica, MA, USA) in DMSO-*d*_6_, with 2.5 parts per million (ppm) as the solvent chemical shift. ^13^C NMR spectra were recorded on a Bruker NMR (100 MHz) spectrometer (Bruker Scientific LLC) in DMSO-*d*_6_, with 39.5 ppm as the solvent chemical shift. Chemical shifts (*δ*) were reported in ppm referenced to the DMSO-*d*_6_ solvent peak. The high-resolution mass spectra (HRMS) were recorded using the (+) ESI mode on a Bruker Daltonics Impact II quadrupole time-of-flight (QTOF) mass spectrometer (gas temperature 200 °C; drying gas N_2_ in a 4 L/min nebulizer at 0.3 bar) at the Mass Spectrometry Research and Education Center of the University of Florida.

### High-resolution liquid chromatography-mass spectrometry (LC–MS) analysis

One milligram of the candidate compound was dissolved in 2 mL of methanol. About 3 µL of this solution was injected with a Dionex UltiMate 3000 autosampler into a 300 μm × 15 cm HPLC C18 column (2 μm, 100 Å Acclaim PepMap; Thermo Fisher Scientific). The HPLC system was the Dionex UltiMate 3000 RSLCnano system. The mobile phase was water with 0.1% formic acid (A) and methanol (B). The flow rate of the loading pump was 25 µL/m, and of the NC pump was 5 µL/m. The gradient was 5% B initially, reaching 99% B at 35–45 min, 99% B at 45–50 min, and 5% B at 55–60 min. The mass spectrometry data were analyzed with a Bruker Daltonics Impact II QTOF spectrometer (in positive mode). The gas temperature was 200 °C. The drying gas was nitrogen with a flow rate of 4 L/min. The nebulizer was at 0.3 bar.

### Rearing mosquitoes

*An. gambiae* (G3 strain) eggs were obtained from BEI Resources (Manassas, VA, USA). Mosquitoes in the insectary were kept at 27 °C, 80% relative humidity, and 12 h day/night cycles. The larvae were fed ground fish food, and the adult mosquitoes were maintained on 8% sucrose solution.

### Culturing of *P. falciparum* gametocytes and ookinetes

As described previously [23], *P. falciparum* (NF54) was cultured in the complete RPMI 1640 medium containing 4% new O+ human red blood cells, 10% human AB+ serum, and 12.5 μg/mL of hypoxanthine in a candle jar at 37 °C. To prepare *P. falciparum* ookinetes, we transferred 5 mL of day-15 cultured *P. falciparum* containing ~ 2% stage V gametocytes into a 15 mL centrifuge tube, followed by centrifugation at 650×*g* for 5 min at RT. The pellet was then resuspended in 2.5 mL of sterile ookinete culture medium (RPMI 1640 medium containing 20% human serum AB+, 50 µg/mL of hypoxanthine, 2 g/L NaHCO_3_). The resuspended cells were transferred into a well of a 12-well plate and incubated at RT on a shaker (20 rpm) for 24 h to generate ookinetes. Finally, cell mixtures of the ookinetes, gametocytes, and asexual-stage *P. falciparum* were collected by centrifugation at 650×*g* for 5 min at RT.

### Analysis of the conversion ratio from gametocytes to ookinetes

The 15- to 17-day-old cultured *P. falciparum* was collected by centrifugation at 500×*g* for 3 min. The pellets were suspended in ookinete culture medium (incomplete RPMI 1640 containing 20% human serum AB+ and 50 µg/mL of hypoxanthine) to obtain 10^5^ gametocytes per microliter. About 1 μL of pulixin (4 mM) in DMSO was added to 99 μL of the ookinete culture medium. After incubation on a shaker (20 rpm) at RT for 18–24 h, the ookinetes and gametocytes were counted using a Giemsa-stained blood smear under a bright-field microscope. The ratios of gametocytes to ookinetes were calculated.

### Inhibition assays of asexual *P. falciparum* proliferation

The 3–5-day cultured iRBCs were mixed with fresh human RBCs (AB+ type) in complete RPMI 1640 to prepare cultures with 0.5% parasitemia and 2% hematocrit. Pulixin was dissolved in DMSO at a concentration of 1 mg/mL and diluted with DMSO to various levels. A 2 µL pulixin solution mixed with 1 mL of cell culture was added to a 24-well plate. The plate was incubated in a candle jar at 37 °C. Approximately 48 h later, the medium was replaced with fresh medium containing the same concentration of pulixin. Parasitemia was recorded at 24, 48, 72, and 96 h post-incubation. The test for each concentration was replicated three times. The concentration that gave half-maximal response (EC_50_) was determined by analyzing the dose–response curve obtained with GraphPad Prism (GraphPad Software, San Diego, CA, USA). The assays were repeated.

### General cytotoxicity assay

A drug can kill a cell or inhibit the cell proliferation through general cytotoxicity. A Vybrant^®^ MTT (3-(4,5-dimethylthiazol-2-yl)-2,5-diphenyl tetrazolium bromide) Cell Proliferation Assay (Thermo Fisher) was used to analyze living cells. The human embryonic kidney 293 (HEK293) cell line was used as the experimental cells. About 20,000 HEK293 cells in 100 µL of culture medium (RPMI 1640 + 2 mM glutamine + 10% fetal bovine serum) were seeded per well in 96-well microplates. After incubation at 37 °C with 5% CO_2_ for 24 h, 1 µL of pulixin in DMSO at various dilutions was added into each well to obtain a final concentration of 0, 1, 3, 10, 30, and 100 µg/mL. Three replicates were conducted for each concentration. Following incubation at 37 °C with 5% CO_2_ for 24 h, 10µL of MTT (5 mg/mL in PBS) was added into each well and incubated for 4 h at 37 °C with 5% CO_2_. All but 25 µL of the medium was removed from the wells, and 100 µL of DMSO was added to each well and incubated at 37 °C for 10 min to dissolve formazan crystals for measurement. Optical density was measured at an absorbance wavelength of 540 nm. The data were analyzed using ANOVA in Prism 8 (GraphPad Software, San Diego, CA, USA). The experiment was performed twice independently.

## Results

### Bioactive fungal extracts against *P. falciparum* transmission

**Fig. 1:**
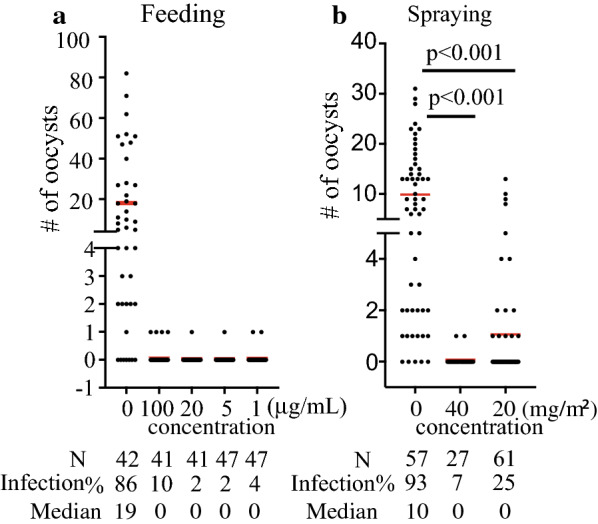
The candidate fungal extract blocks *P. falciparum* transmission through feeding or spraying. **a** The fungal extract (GFEL-12E6) significantly inhibited *P. falciparum* infection in mosquito midguts in the SMFA. The experiment was independently conducted three times and the results were consistent. **b** Exposure to the fungal extract significantly inhibited *P. falciparum* infection in mosquito midguts. The fungal extract in acetone was sprayed onto cups and dried. Mosquitoes were placed in the cup for 24 h before the SMFA. This showed one experiment, and the experiment was independently conducted three times, and the results were consistent. The red line indicates the median number of oocysts in mosquitoes in each treatment. The bold lines at zero oocysts indicate many mosquitoes with zero oocysts. N: the number of mosquitoes in the group. Infection (%): the percentage of infected mosquitoes. Median: the median number of oocysts in mosquito midguts

Because FREP1–parasite interaction facilitates malaria transmission, we used an ELISA-based approach [18] to screen 1232 ethyl acetate extracts (40 µg/mL) in the global fungal extract library (GFEL) [20] that prevented FREP1 protein from binding to *P. falciparum*-infected cell lysate. The extracts that inhibited 90% of the FREP1**–**parasite interaction were further analyzed for their activity in blocking malaria transmission with SMFA [18]. Here, we focused on one fungal extract, GFEL-12E6 (GFEL plate 12, row E, column 6), because it completely inhibited transmission of *P. falciparum* to *An. gambiae* at 1 µg/mL and was more active than the other candidates. A series of dilutions, from 100 to 1 µg/mL, of the GFEL-12E6 crude fungal extract inhibited the transmission of *P. falciparum* to *An. gambiae* (Fig. 1a). This fungal extract at 1 µg/mL rendered 45 out of 47 mosquitoes free of *P. falciparum* infection, and 2 out of 47 mosquitoes had only one oocyst. In the control group, about 86% of mosquitoes were infected with *P. falciparum*, i.e. 36 out of 42 mosquitoes had oocysts in their midguts.

Spraying agents to block malaria transmission is a novel approach, proposed and tested by the corresponding author Dr. Jun Li’s group in 2015 and confirmed by another laboratory in 2019 [19]. This method will significantly facilitate the future application of antimalarial agents. We examined the effect of GFEL-12E6 sprays on malaria transmission to mosquitoes. The GFEL-12E6 extract in acetone was sprayed on the inner surface of paper cups. After drying, about 100 mosquitoes were placed in the treated cups for 24 h and then fed *P. falciparum*-infected blood. The engorged mosquitoes were maintained in a new clean cup without any fungal extract spray. The negative controls were cups treated with acetone only. Results showed that significantly fewer *P. falciparum* oocysts developed in the mosquitoes pre-exposed to GFEL-12E6 than in those in the control (Fig. 1b). Spraying with the fungal extract inhibited *P. falciparum* infection in mosquitoes. As little as 20 mg/m^2^ of GFEL-12E6 was capable of significantly reducing (*p* < 0.001) *P. falciparum* infection load in mosquitoes. The median number of oocysts and the infection prevalence rate were 10 and 93%, respectively, in the control group. After exposure to GFEL-12E6 extract spray at 20 mg/m^2^, the median number of oocysts was 0 and infection prevalence was 25% (Fig. 1b). This inhibition was dose-dependent. Spraying with the 40 mg/m^2^ extract resulted in 93% of mosquitoes being free from *P. falciparum* infection (Fig. 1b).

### Identification of the candidate fungal species

**Fig. 2:**
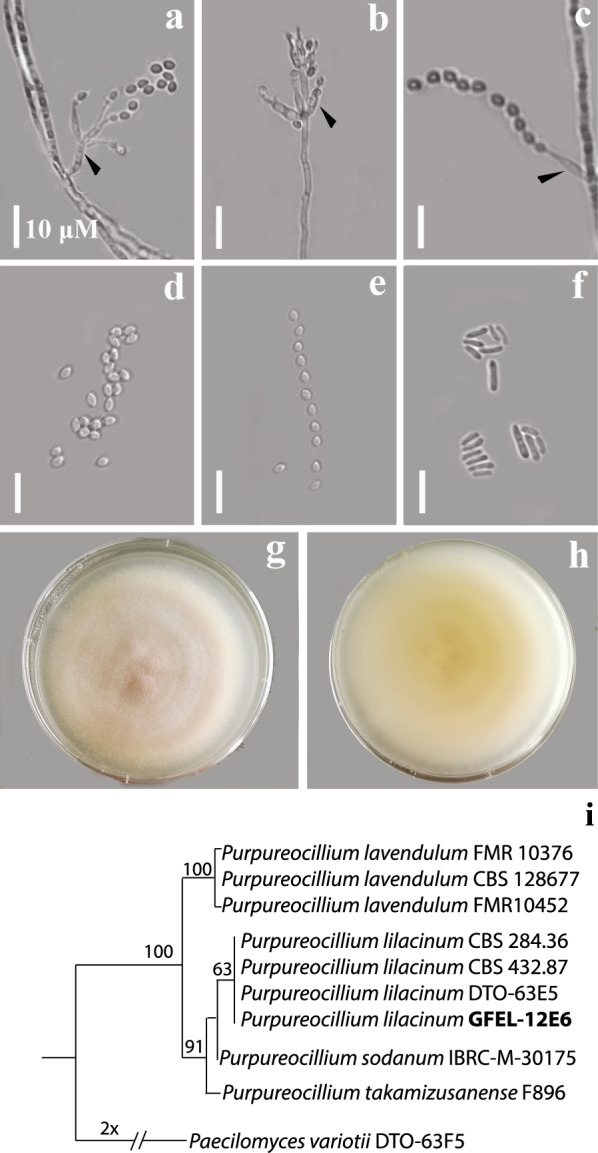
The candidate fungus was identified as *Purpureocillium lilacinum*. Colonies for observation were grown on potato dextrose agar (PDA) medium plates for 7–15 days at 25 °C. **a** Short conidiophores; **b** long conidiophores; **c** solitary phialide-producing catenate conidia. Arrows in **a**, **b**, **c** point to phialides. **d**, **e** Typical sub-globose conidia; **f** cylindrical conidia; **g** colony surface on PDA medium plate (top); **h** colony reverse on PDA (bottom). Scale bar: 10 μM. **i** A maximum parsimony tree was constructed based on ITS sequences, and bootstrap values above 50% are indicated at the nodes

Since GFEL-12E6 is functional in limiting malaria transmission, we undertook further studies to identify the species of this candidate fungus. The morphology of the candidate fungus was examined under a microscope. The conidiophores growing from the aerial mycelium were short and branched without a specific pattern, with 1–4 phialides per branch (Fig. 2a). In contrast, the conidiophores rising from the superficial mycelium were very long and bore verticillate branches with whorls of 2–4 phialides (Fig. 2b). Phialides were 2.5–3 × 7–9.5 μm in dimension, with a swollen basal portion tapering into a distinct neck about 1 μm in length (Fig. 2a, b). Phialides that produced *Acremonium*-like conidiophores were very long (up to 30 μm) and solitary (Fig. 2c). Conidia were in long dry chains, subglobose, 2–3 × 3–4 μm, smooth-walled to slightly roughened, hyaline, and purple in mass (Fig. 2d, e). Some conidia were cylindrical and were 1.5–2.5 × 2.0–13.5 μm in dimension (Fig. 2f). Colonies on potato dextrose agar (PDA) medium plates attained a size of 50 mm and 65 mm in diameter after 15 days and 30 days of incubation, respectively, at 25 °C. Colonies consisting of a dense basal felt were white at the beginning, later becoming purple in color (Fig. 2g). From the reverse side of the plate, the colony appeared to be light yellow (Fig. 2h). The morphology of this fungus was similar to that of *Purpureocillium lilacinum* re-named from *Paecilomyces lilacinu*m [24].

To further identify the species, the conserved intergenic space of the fungal genome was PCR-amplified with ITS1/ITS4 primers [18] and sequenced. Phylogenetic analysis of the ITS (Fig. 2i) showed that the candidate fungus GFEL-12E6 and other *Purpureocillium* species were in the same monophyletic clade with 100% maximum parsimony (MP). The candidate fungus was clustered together with the identified *Purpureocillium lilacinum* strains (63% MP). The phylogenetic analysis confirmed that the candidate fungus GFEL-12E6 was *Purpureocillium lilacinum*.

### Isolation and identification of fungal metabolites

**Fig. 3:**
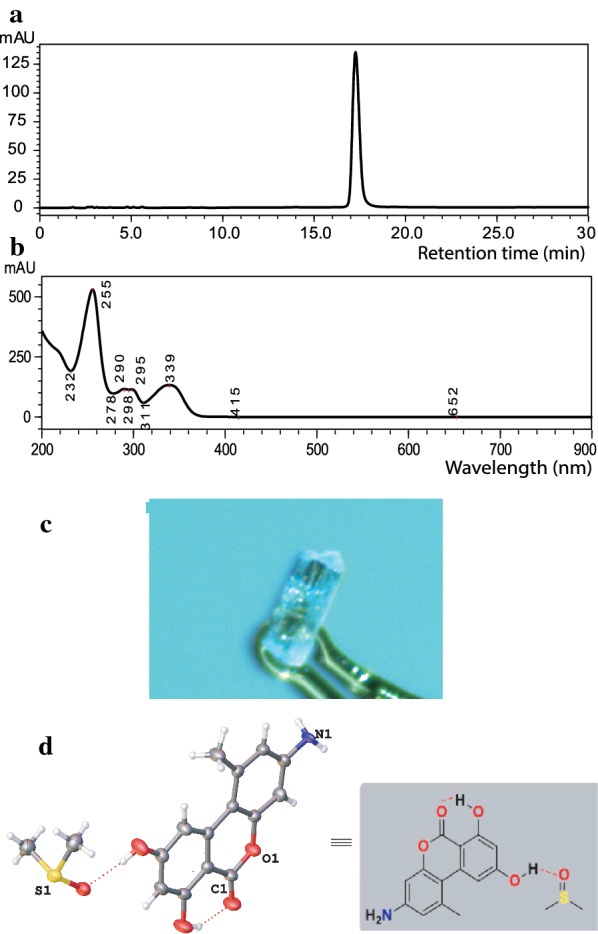
The spectrum for the isolated pure compound from GFEL-12E6. **a** The HPLC profile of the pure compound shows one peak. **b** The absorbance spectrum of the purified compound. **c** The crystal of pulixin. **d** The structure of pulixin. Since DMSO was used as a solvent to grow a crystal, DMSO formed a hydrogen bond with pulixin

GFEL-12E6 in methanol was fractioned by preparative TLC, and the bioactivity of each fraction was detected by SMFA. One bioactive fraction was further purified by HPLC to obtain a pure compound at 17.2 min retention time (Fig. 3a). The active compound was named “pulixin.” UV–visible absorbance spectra showed characteristic peaks at 255, 290, 295, and 339 nm (Fig. 3b).

Pulixin was also crystallized from DMSO by slow evaporation at RT (Fig. 3c), yielding a colorless solid crystal. The structure of the crystal was determined by X-ray crystallography. In the crystal, an interstitial DMSO molecule was hydrogen-bonded to pulixin. Based on the X-ray structure determination, pulixin was identified as 3-amino-7,9-dihydroxy-1-methyl-6*H*-benzo[*c*]chromen-6-one (Fig. 3d). The crystal data were submitted to the Cambridge Crystallographic Data Centre with the deposition number 2005130.

We also determined the molecular mass of pulixin using HR-ESI-mass spectrometry to confirm its identity in the bulk extract. An [M+H]^+^ ion at m/z 258.0764 was observed, matching the calculated mass of 258.0766 amu (Additional file 1: Figure S1). In addition, NMR was used to confirm the structure of the active compound. ^1^H-NMR data (Additional file 1: Figure S2) confirmed the presence of a specific hydrogen-bonded hydroxyl group (*δ* = 11.76 ppm), an amino group (*δ* = 10.61 ppm), and a methyl group (*δ* = 2.67 ppm). The ^13^C-NMR data (Additional file 1: Figure S3) were consistent with the presence of an ester keto group (*δ* = 165.4 ppm), two hydroxyl groups bearing aromatic carbon atoms (*δ* = 164.6 and 164.0 ppm), one amino group attached to an aromatic carbon atom (*δ* = 152.6 ppm), and a methyl carbon atom attached to the aromatic ring (*δ* = 25.2 ppm). Table 1 summarizes the NMR data. Collectively, the data for the structure of pulixin unambiguously confirmed it to be 3-amino-7,9-dihydroxy-1-methyl-6*H*-benzo[*c*]chromen-6-one.

**Table 1:** ^1^H NMR and ^13^C NMR data of antimalarial drug 3-amino-7,9-dihydroxy-1-methyl-6*H*-benzo[*c*]chromen-6-one (*δ* in ppm, *J* in Hz and NMR solvent DMSO-*d*_*6*_)

Position	^1^H NMR data (400 MHz)	^13^C NMR data (100 MHz)	Structure
1	–	138.2	**1**
2	6.69 (d, *J* = 2.1 Hz, 1H)	108.9	
3	–	152.6	
4	6.61 (d, *J* = 2.2 Hz, 1H)	100.8	
5	–	158.4	
6	–	101.6	
7	–	164.6	
8	6.34 (d, *J* = 1.2 Hz, 1H)	97.3	
9	–	164.0	
10	7.21 (s, 1H)	104.3	
11	–	138.0	
12	–	117.5	
13	–	165.4	
–CH_3_	2.67 (s, 3H)	25.1	
–NH_2_	10.61 (s, br, 2H)		
–OH	11.76 (s, 1H), 5.74 (s, 1H)		

### Pulixin prevented FREP1 from binding to *P. falciparum* and blocked *P. falciparum* transmission to *An. gambiae*

**Fig. 4:**
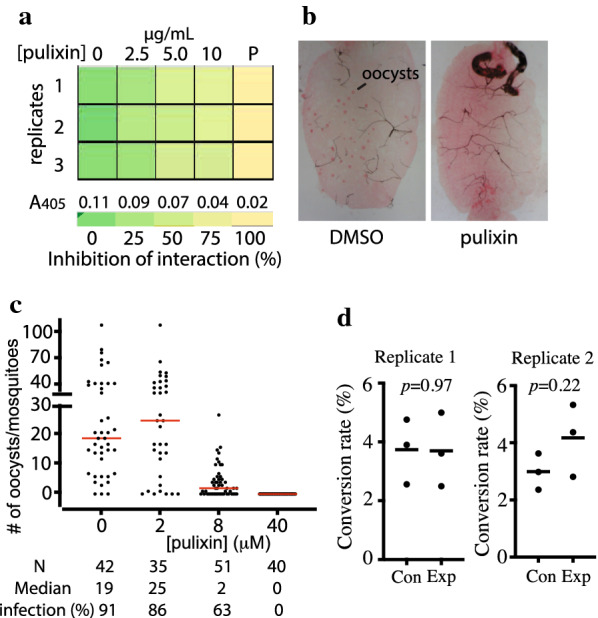
Pulixin inhibited the FREP1–*P. falciparum* interaction and blocked malaria transmission. **a** ELISA results showed that pulixin inhibited the interaction between FREP1 and *P. falciparum*-infected cell lysate and that the inhibition was dose-dependent. P: The positive control using the heat-inactivated FREP1 that did not interact with parasites. **b** The midguts of pulixin-treated mosquitoes had fewer oocysts than those of the control (DMSO) mosquitoes. Red dots inside the midguts are oocysts. **c** Pulixin inhibited the transmission of *P. falciparum* to *An. gambiae* in a dose-dependent manner. This experiment was conducted twice independently, and the results were similar. Each dot represents the number of oocysts in an experimental mosquito. Red lines show the median number of oocysts. N: number of mosquitoes. Median: median number of oocysts. Infection (%): percentage of infected mosquitoes. **d** Pulixin did not inhibit the formation of ookinetes. The assay was conducted twice independently. Each repeat had three replicates in the experimental and control groups. The conversion rate was defined as $\frac{number\;of\;ookinetes}{{number\;of\;gametocytes}} \times 100$. Black lines depict the means of conversion rates. Con: control groups with DMSO in culture; Exp: experimental groups with 40 μM of pulixin in culture

We determined the activity of pulixin in preventing FREP1 from binding to parasite-infected cell lysate using ELISA assays. The A_405_ values differed among wells with different concentrations of pulixin (Fig. 4a). As the pulixin concentration increased from 0 to 10 µg/mL, less FREP1 was retained. DMSO (1%, v/v) without the compound was used as a non-inhibition control. Heat-inactivated FREP1, which did not bind to the parasite-infected cell lysate, was used to replace FREP1 as the 100% inhibition control (labeled as P in Fig. 4a). Based on the A_405_ values, inhibition rates at different concentrations were calculated. The results showed that pulixin inhibited the interaction between the FREP1 protein and *P. falciparum*-infected cell lysate, and the inhibition was dose-dependent (Fig. 4a). At a concentration of 5 µg/mL, pulixin inhibited about 50% of the interaction between the FREP1 protein and *P. falciparum*-infected cell lysate.

Next, we analyzed the effects of pulixin on *P. falciparum* infection in mosquitoes. Pure pulixin in DMSO was mixed with *P. falciparum*-infected blood at concentrations from 0 to 40 μM and fed to *An. gambiae* using SMFA. The midguts in the experimental groups contained fewer oocysts, stained as red dots, than those in the control (DMSO; Fig. 4b). Pulixin completely inhibited malaria transmission at a concentration of 40 μM, and inhibition activity decreased as the level of pulixin decreased (Fig. 4c). EC_50_, defined as the concentration of a compound that inhibits 50% of infection intensity (the number of oocysts per mosquito), in experimental mosquitoes compared to that of the control group was 11 μM, calculated using a serial dilution of samples with an LC_50_ calculator [25]. We analyzed the activity of pulixin again after storing it in the laboratory at RT for 6 months and obtained a similar result.

Pulixin at a level of 40 μM completely blocked malaria transmission. Therefore, we examined whether 40 μM of pulixin inhibited conversion of gametocytes to ookinetes. Results showed no significant difference in gametocyte transformation rates between the control and pulixin-treated samples (*p* > 0.2; Fig. 4d), suggesting that pulixin did not affect the conversion of gametocytes to ookinetes.

### Pulixin inhibited the development of the asexual *P. falciparum*

**Fig. 5:**
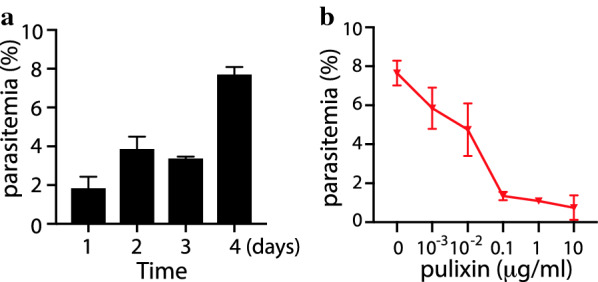
Pulixin was able to inhibit the development of the asexual-stage *P. falciparum* in blood. The test for each concentration was replicated three times and the assays were repeated. The profiles show the means and standard errors. **a** Parasitemia at days 1, 2, 3, and 4 after inoculation with *P. falciparum*-infected blood without pulixin. Significantly more (*p* < 0.001) parasite-infected cells at day 4 comparing to day 1. **b** Parasitemia on day 4 after incubation with different concentrations of pulixin. Significantly fewer *P. falciparum*-infected cells were observed when the concentration of pulixin was greater than 0.01 μg/mL (*p* < 0.05), compared to the control (0 μg/mL pulixin)

First, we analyzed the parasitemia to determine the parasite proliferation profile over 4 days. *P. falciparum*-infected blood was added to the fresh medium with uninfected red blood cells to obtain 0.5% parasitemia in 2% hematocrit. The culture was incubated for 4 days, with medium changed on day 2, and the parasitemia was analyzed every day. The results (Fig. 5a) showed that parasitemia on day 4 was significantly higher (*p* < 0.001) than that on day 1, and almost all infected cells were at the asexual stage. This result is consistent with the *P. falciparum* 48-h asexual replication cycle. Based on this result, we examined the activity of pulixin in inhibiting the development of asexual-stage *P. falciparum* on the fourth day after inoculation. The results showed that the inhibitory effect of pulixin on asexual-stage *P. falciparum* development was dose-dependent, and inhibition increased as the pulixin concentration increased (Fig. 5b). The EC_50_ of pulixin in inhibiting the development of the asexual-stage *P. falciparum* was 0.012 μg/mL, or 47 nM.

### Pulixin did not show general cytotoxicity to human cells

**Fig. 6:**
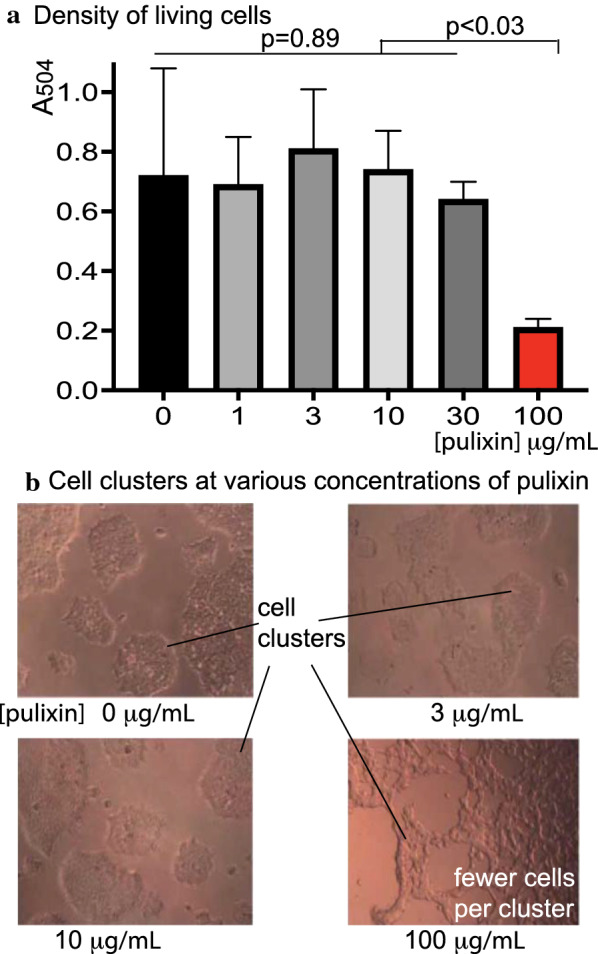
Pulixin did not show significant cytotoxicity to the human embryonic kidney 293 cell line at a concentration of 30 µg/mL or lower. **a** The cytotoxic effects of pulixin on human embryonic kidney 293 (HEK293) cell proliferation at varying concentrations (0–100 µg/mL) were measured with MTT assays. No significant difference (*p* = 0.89) was observed when the concentration of pulixin was 30 µg/mL or lower. The density of living cells was significantly lower when pulixin reached 100 µg/mL, compared to the other concentrations (*p* < 0.03). The test for each concentration was replicated three times. The data were analyzed using ANOVA. **b** Cells were observed under bright-field microscopy. Consistent with MTT assays, many fewer cells were observed when the concentration of pulixin reached 100 µg/mL than under other concentrations, e.g., pulixin ≤ 30 µg/mL

Following confirmation that pulixin inhibited both the sexual and asexual stages of *P. falciparum*, we analyzed its general cytotoxicity to cells using MTT assays. These assays measured the density of living cells. Human embryonic kidney 293 cells (HEK293) were incubated with pulixin at different concentrations. No significant difference in the density of living cells was observed in cultures with a pulixin level ranging from 0 to 30 µg/mL (116 µM; *p* = 0.88; Fig. 6a) suggesting that pulixin did not have significant cytotoxic effects on HEK293 cells at these concentrations. When the level of pulixin was increased to 100 μg/mL, a significant reduction in cell density occurred (*p* < 0.05; Fig. 6a), and far fewer cells per cluster were observed under a microscope (Fig. 6b) compared to the other groups.

## Discussion

Insecticide-resistant mosquitoes, drug-resistant *Plasmodium* parasites, and the lack of malaria vaccines challenge the current efforts to control malaria. Malaria transmission depends on infected mosquitoes. Blocking *Plasmodium* transmission to mosquitoes will break the malaria infection cycles, and natural fungal metabolites are excellent sources of potential malaria-blocking drugs [18].

The discovery of the molecular mechanism of the FREP1–parasite interaction makes high-throughput screening of drugs against malaria transmission possible [12]. Targeting the mosquito FREP1-mediated *Plasmodium* transmission pathway, we screened fungal extracts from our newly established global fungal library [20] for inhibition of the FREP1–*Plasmodium* lysate interaction. Those extracts showing positive activity have the potential to inhibit malaria transmission. Among the positive hits, the crude extract from a *Purpureocillium lilacinum* strain was able to completely block *P. falciparum* transmission at a concentration of 1 μg/mL. Strikingly, when mosquitoes were pre-exposed to this extract through contact, they became resistant to *P. falciparum* infection. These data are consistent with a recent paper that showed that anopheline mosquitoes pre-exposed to atovaquone became resistant to *P. falciparum* transmission [19]. Spraying bioactive compounds indoors, on bed nets, or on clothes to stop malaria transmission would simplify their application.

We also isolated and identified pulixin, a novel compound that inhibited *Plasmodium* development at multiple stages, including the sexual stage of *P. falciparum* transmitted to mosquitoes and the asexual stage infected blood. Pulixin is a benzo[*c*]chromen-6-one derivative possessing one amino and two hydroxyl functional groups. Molecules bearing benzo[*c*]chromen-6-one are biologically and pharmaceutically essential and are extensively distributed in nature [26, 27]. Although several natural and synthetic molecules carry the benzo[*c*]chromen-6-one moiety, pulixin is novel and has not been hitherto synthesized and isolated from any sources. The structure of pulixin is related to that of alternariol, with the C-3 OH in alternariol substituted with the C-3 NH_2_ in pulixin. Alternariol was shown to be toxic to female mice at a concentration of 400 mg/kg injected intraperitoneally, e.g., 1.5 mM [28]. Our results showed that pulixin did not inhibit proliferation of or present significant toxicity to the human kidney cell line HEK293 at a concentration of 116 μM or less. While 40 μM of pulixin completely blocked transmission of *P. falciparum* to *An. gambiae*, it did not inhibit the conversion of gametocytes to ookinetes. Notably, the EC_50_ of pulixin in inhibiting malaria transmission to mosquitoes was 17 μM. Thus, the inhibition effect of pulixin on malaria transmission is unlikely to be through general cytotoxic activity. On the other hand, pulixin inhibited the FREP1–parasite interaction at an EC_50_ of 5 μg/mL (~ 20 μM), consistent with the hypothesis of targeting the FREP1-mediated transmission pathway to block malaria transmission. Since this pathway is conserved across *Plasmodium* species [13], *Purpureocillium lilacinum* extract and pulixin are expected to inhibit other malaria pathogens such as *P. vivax* carried by *Anopheles* mosquitoes. The structure of pulixin suggests that it is stable, which is consistent with our observation that pulixin did not lose any activity after storing it in a laboratory at RT for more than 6 months. To develop a commercial spray product from the pulixin, measurements of its stability in the environment and toxicity to plants, birds, and fish are needed.

It was a surprise that pulixin also inhibited the reproduction of asexual *P. falciparum* at an EC_50_ of 47 nM; this activity is comparable to that of one of the most powerful antimalarial drugs, artemisinin, whose EC_50_ is 17.3 nM [29]. While artemisinin does not inhibit malaria transmission, pulixin can completely block it. Another antimalarial drug recommended by WHO, primaquine, blocks *P. falciparum* transmission at a dose of 0.5–0.75 mg/kg (2–3 μM) [30]. However, primaquine has adverse effects, including causing hemolytic anemia in G6PF-deficient patients. The molecular mechanism of pulixin inhibiting malaria proliferation in erythrocytes is unknown at this time.

## Conclusions

We screened a sizable fungal extract library and discovered a fungus, *Purpureocillium lilacinum*, that produces small molecules to block malaria transmission. Moreover, we isolated and identified the structure of the bioactive compound, pulixin, in the fungal extract. It is a novel natural compound that inhibits asexual *Plasmodium* replication and sexual *Plasmodium* transmission. *Purpureocillium lilacinum* extract and pulixin are excellent new tools for malaria control.

## Supplementary Information

**Additional file 1: Figure S1.** Identification of the candidate compound through the mass spectrometric profile of pulixin showing a mass of 258.0764, which matched the calculated mass. **Figure S2.** The H-NMR profile of pulixin confirmed its structure. **Figure S3.** The C-NMR profile of pulixin was consistent with the proposed structure.

## Data Availability

The data for the pulixin crystal structure have been deposited in the Cambridge Crystallographic Data Centre (deposition number 2005130). The ITS sequence of GFEL-12E6 is available at GenBank with the accession number MW388000.
