# Supplementary material for: A novel fungal metabolite inhibits Plasmodium falciparum transmission and infection
Source: Parasit Vectors. 2021 Mar 24;14:177. doi: 10.1186/s13071-021-04677-7 (PMC7992847; doi:10.1186/s13071-021-04677-7)
Supplement: Supplementary file 1 — Additional file 1: Figure S1. Identification of the candidate compound through the mass spectrometric profile of pulixin showing a mass of 258.0764, which matched the calculated mass. Figure S2. The H-NMR profile of pulixin confirmed its structure. Figure S3. The C-NMR profile of pulixin was consistent with the proposed structure. [file 13071_2021_4677_MOESM1_ESM.pdf]

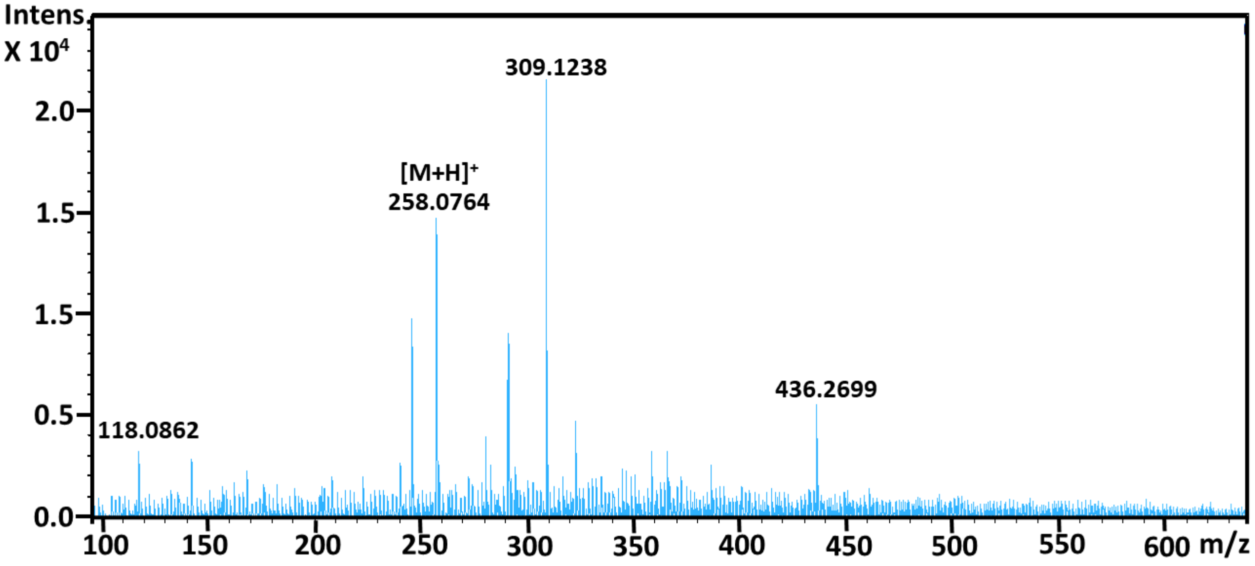

Fig S1: Identification of candidate compound through the mass spectrometry profile of pulixin, showing the mass of 258.0764, which matched the calculated mass.

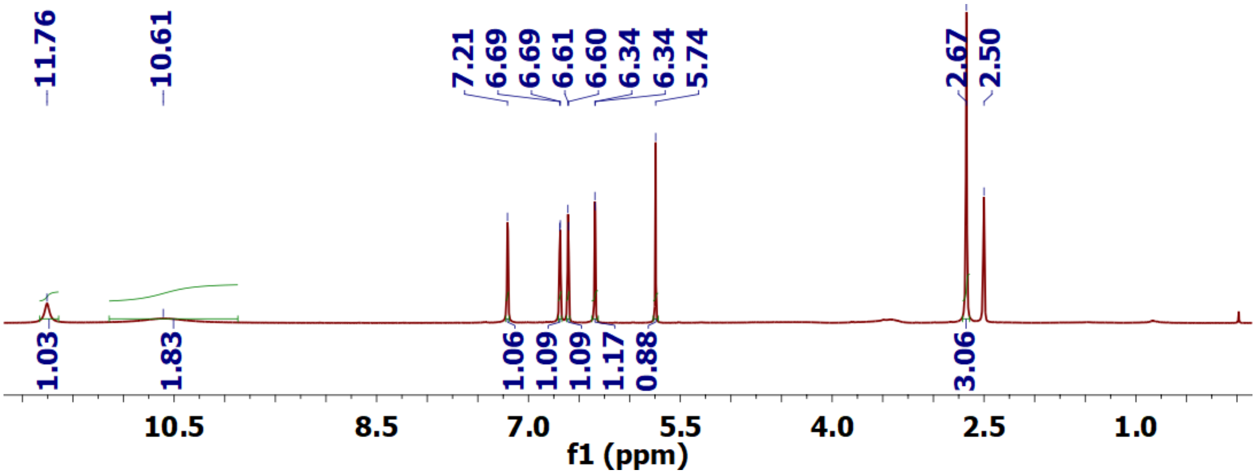

Fig S2: The H-NMR profile of pulixin confirmed the structure of pulixin.

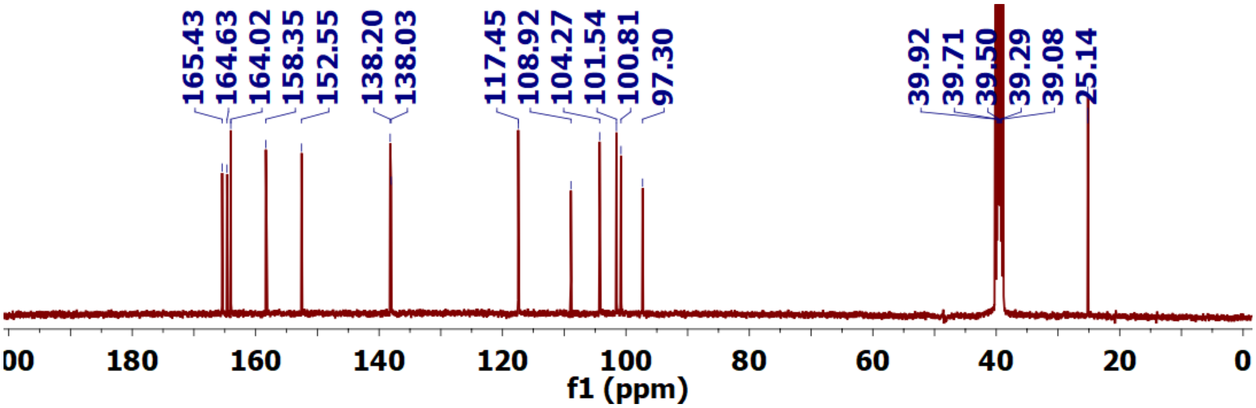

Fig S3: The C-NMR profile of pulixin was consistent with the proposed structure.
